# Influence of conservation agriculture-based production systems on bacterial diversity and soil quality in rice-wheat-greengram cropping system in eastern Indo-Gangetic Plains of India

**DOI:** 10.3389/fmicb.2023.1181317

**Published:** 2023-07-05

**Authors:** Rakesh Kumar, Jaipal Singh Choudhary, Sushanta Kumar Naik, Surajit Mondal, Janki Sharan Mishra, Shish Pal Poonia, Saurabh Kumar, Hansraj Hans, Sanjeev Kumar, Anup Das, Virender Kumar, Bhagwati Prasad Bhatt, Suresh Kumar Chaudhari, Ram Kanwar Malik, Peter Craufurd, Andrew McDonald, Sonam Rinchen Sherpa

**Affiliations:** ^1^ICAR Research Complex for Eastern Region, Patna, Bihar, India; ^2^ICAR Research Complex for Eastern Region, Farming System Research Centre for Hill and Plateau Region, Ranchi, Jharkhand, India; ^3^ICAR-Directorate of Weed Research, Jabalpur, Madhya Pradesh, India; ^4^Cereal Systems Initiative for South Asia (CSISA)-CIMMYT, Patna, India; ^5^International Rice Research Institute, Los Banos, Philippines; ^6^ICAR Natural Resource Management Division, New Delhi, India; ^7^Stratigic Research Team, CIMMYT, Kathmandu, Nepal; ^8^Soil and Crop Sciences Section, School of Integrative Plant Sciences, Cornell University, Ithaca, NY, United States

**Keywords:** conservation agriculture, metagenomics, soil quality, earthworm, bacterial diversity, DNA sequencing, rice-wheat-greengram

## Abstract

**Introduction:**

Conservation agriculture (CA) is gaining attention in the South Asia as an environmentally benign and sustainable food production system. The knowledge of the soil bacterial community composition along with other soil properties is essential for evaluating the CA-based management practices for achieving the soil environment sustainability and climate resilience in the rice-wheat-greengram system. The long-term effects of CA-based tillage-*cum*-crop establishment (TCE) methods on earthworm population, soil parameters as well as microbial diversity have not been well studied.

**Methods:**

Seven treatments (or scenarios) were laid down with the various tillage (wet, dry, or zero-tillage), establishment method (direct-or drill-seeding or transplantation) and residue management practices (mixed with the soil or kept on the soil surface). The soil samples were collected after 7 years of experimentation and analyzed for the soil quality and bacterial diversity to examine the effect of tillage-*cum*-crop establishment methods.

**Results and Discussion:**

Earthworm population (3.6 times), soil organic carbon (11.94%), macro (NPK) (14.50–23.57%) and micronutrients (Mn, and Cu) (13.25 and 29.57%) contents were appreciably higher under CA-based TCE methods than tillage-intensive farming practices. Significantly higher number of OTUs (1,192 ± 50) and Chao1 (1415.65 ± 14.34) values were observed in partial CA-based production system (*p ≤ 0.05*). Forty-two (42) bacterial phyla were identified across the scenarios, and *Proteobacteria*, *Actinobacteria*, and *Firmicutes* were the most dominant in all the scenarios. The CA-based scenarios harbor a high abundance of *Proteobacteria* (2–13%), whereas the conventional tillage-based scenarios were dominated by the bacterial phyla *Acidobacteria* and *Chloroflexi* and found statistically differed among the scenarios (*p* ≤ 0.05). Composition of the major phyla, i.e., *Proteobacteria, Actinobacteria*, and *Firmicutes* were associated differently with either CA or farmers-based tillage management practices. Overall, the present study indicates the importance of CA-based tillage-*cum*-crop establishment methods in shaping the bacterial diversity, earthworms population, soil organic carbon, and plant nutrient availability, which are crucial for sustainable agricultural production and resilience in agro-ecosystem.

## 1. Introduction

Soil health is critical to the success of agriculture for meeting the increasing demand for food fiber, and ensuring sustainable food production (Mondal et al., 2020; Kumar et al., 2022a). It is an important factor in determining productivity and fertility of the soil, which ultimately impacts crop yield and quality. Maintaining healthy soil is crucial for ensuring sustainable agricultural practices. The Indo-Gangetic Plains (IGP) of South Asia are a significant contributor to the food production of India and other surrounding countries. The region is characterized by high population density and intensive agricultural activities, making it one of the most productive agricultural areas in the world (Saurabh et al., 2021). In IGP, middle Indo-Gangetic Plains (MIGP), covering eastern Uttar Pradesh and Bihar are endowed with rich and diverse natural resources but have lower crop productivity and *per capita* income (Kumar et al., 2020). The rice-wheat system is a prevalent cropping pattern in the Indo-Gangetic Plains (IGP) of South Asia and covers a large portion (~40%) of the agricultural area in the region (Panigrahy et al., 2010). Degradation of soil is a major concern in the past few decades in the MIGP, which is partially driven by intensive tillage practices followed in conventional agriculture (Mishra et al., 2021). Practices like intensive tillage operations, absence of crop residue, imbalance use of chemical fertilizers and lack of crop rotation in conventional farming lead to deterioration of soil (Samal et al., 2017). Soil degradation will be more intense in the future due to the increased intensity of conventional tillage (CT), consequences of climate change and more pressure on unit areas for food production (Mishra et al., 2022). Hence, adopting good soil-conservation practices, such as minimal soil disturbance (Zhang et al., 2015), retention of crop biomass (Devi et al., 2017), soil cover (Mondal et al., 2021), sufficient use of manures (Kumar et al., 2021) and crop diversification are important aspects for long-term sustainability of agriculture.

The upper soil horizon is substantially disrupted by CT, resulting in a homogeneous layer of soil with fairly consistent physical properties and nutrient distribution. CT practices also have impacts on chemical characteristics of the soil and may also lead to changes in bacterial, fungal, and arthropod diversity as well as abundance in the soil (Xin et al., 2018; Choudhary et al., 2020; Sun et al., 2020). In comparison to CT, minimal or no-tillage (NT) practices have a profound improvement in soil organic carbon (SOC), nutrient status, water holding capacity, soil erosion, and equilibrium in soil moisture and temperature changes (Guo et al., 2016; Schmidt et al., 2018; Sun et al., 2018). Therefore, CA practices create a more friendly and laminated environment for proliferation of soil microorganisms and other macro-and micro-fauna (Dumanski and Peiretti, 2013). In totality, adoption of conservation agricultural (CA) practices has an excellent possibility of increasing soil quality with increased carbon substrates, enhanced microbial diversity and activity, improved soil structure, and better use efficiencies of agricultural inputs (Ghosh et al., 2015; Li et al., 2018).

Soil resilience is a crucial aspect of sustainable agriculture production systems. The physical, chemical, and biological properties of soil determine its resilience and its ability to withstand changes and maintain its fertility (Brejda et al., 2000). Soil microorganisms are a vital component of soil biological properties and play a key role in nutrient cycling and soil organic matter decomposition (Kibblewhite et al., 2008). They help maintain soil fertility, improve soil structure, and contribute to the overall health and productivity of the soil. Maintaining soil resilience through practices such as soil conservation, crop rotation, and the use of organic matter is essential for sustainable agriculture and ensuring long-term food security. Plants with their associated microorganisms form an assemblage of species, referred to as “holobiont.” Plant-beneficial microorganisms assist plant holobiont to adapt to biotic and abiotic environmental variations thus, leading to enhanced plant fitness under unfavorable conditions (Hassani et al., 2018; Lyu et al., 2021). They also respond to environmental changes thus, creating soil resilience to climate change (Kennedy and Smith, 1995). Therefore, soil microbial community composition needs to be studied, and our understanding of its functions in agro-ecosystems needs to be augmented. Plentiful studies have been conducted to understand the effect of different agricultural management practices on soil characteristics, soil carbon pools, and productivity and profitability in South Asia and their part in reducing GHGs and adapting to climatic threats (Mbuthia et al., 2015; Shekhawat et al., 2016; Nandan et al., 2018; Singh V. K. et al., 2018; Jat et al., 2020). Despite the many studies conducted on the impacts of crop establishment, tillage, and residue management on soil properties and crop productivity in South Asia, there is a limited understanding of the effects of these practices on microbial community composition and structure in the Middle Indo-Gangetic Plains (MIGP) of the region. The microbial community is a critical component of soil health and fertility, and plays a crucial role in nutrient cycling and soil organic matter dynamics. Further research on the impact of different management practices on the microbial community in the MIGP is needed to better understand their role in building soil resilience towards environmental changes and supporting sustainable agricultural practices in the region. Understanding the relationships between management practices, microbial community composition, and soil properties will provide valuable insights into how to optimize agricultural practices for increased food security and environmental sustainability in the MIGP.

Globally, several studies have been concentrated on crop establishment, tillage and crop residue effects on taxonomic diversity of bacteria but findings of these experiments have varied on soil microbial community structure and abundance (Lienhard et al., 2014; Guo et al., 2016; Wang et al., 2017; Xia et al., 2019; Sun et al., 2020). Nevertheless, previous studies have focused primarily on specific aspects, such as tillage and crop cover, when investigating the microbial structure within CA-based management practices. However, there has been a limited number of studies that have taken into account a broader range of CA practices (Choudhary et al., 2020). Due to the interdependencies, chemical and biological properties are simultaneously affected by various management practices. The effects of tillage-based management practices on soil microbial diversity and abundance can vary depending on the specific tillage method used, the soil type, and the environmental conditions. Some studies have found that tillage practices can lead to increased soil microbial diversity and abundance, while others have shown a decrease or no change (Yin et al., 2010; Guo et al., 2016; Wang et al., 2017; Hartman et al., 2018; Xia et al., 2019). It is important to note that the impact of tillage practices on soil microbial communities can be complex and may change over time, making it challenging to generalize the effects across different systems. Earthworm activity, soil chemical properties, and bacterial communities are important components of the plant–soil-microbial system, and understanding the complex relationships among them is crucial for managing agroecosystems sustainably. Earthworms play a key role in soil structure and nutrient cycling, while changes in soil chemical properties can influence plant growth and health, as well as soil microbial communities. Bacterial communities, in turn, can play a critical role in nutrient cycling and decomposition processes. By studying the interactions between these components, scientists can gain a deeper understanding of how to manage agroecosystems in a way that promotes soil health and sustainable crop production. Considering the above facts and gaps, a long-term experiment was initiated for studying influence of long term tillage-*cum*-crop establishments and crop cover on changes in soil microbial community structure, abundance and soil quality parmeters under sub-tropical humid climate of eastern Indo-Gangetic Plains (EIGP).

## 2. Materials and methods

### 2.1. Site description and experimental design

A long-term experiment was initiated in 2015 at experimental farm of the Indian Council of Agricultural Research – Research Complex for Eastern Region, Patna (25 °24.912’ N and 85 °03. 536′ E). Climate is sub-tropical humid with an average annual rainfall of 1,130 mm. January and May are the coldest and hottest months with average minimum and maximum temperatures of 7–9°C and 36–41°C, respectively. Relative humidity varies from 60 to 90% throughout the year. The experiment was carried out with seven treatments referred as ‘scenarios’ (sc) in a randomized block design and replicated thrice. The dimension of plots was 20 m × 8 m. Seven tillage-*cum*-crop establishment methods (TCE) for a rice-wheat-greengram system were: (1) puddled random transplanted rice (RPTR) – conventional till (CT) broadcast wheat (BCW) – zero-till greengram (ZTG) [RPTR-CTBCW-ZTG]; (2) puddled line transplanted rice (LPTR) – CT drilled wheat (CTW) – ZTG [LPTR-CTW-ZTG]; (3) puddled machine transplanted rice (CTMTR) – zero-till wheat (ZTW) – ZTG [CTMTR-ZTW-ZTG]; (4) zero-till machine transplanted rice (ZTMTR) – ZTW – ZTG [ZTMTR-ZTW-ZTG]; (5) system of rice intensification (SRI) – system of wheat intensification (SWI) – ZTG [SRI-SWI-ZTG]; (6) CT direct-seeded rice (CTDSR) – ZTW – ZTG [CTDSR-ZTW-ZTG]; and (7) zero-till DSR (ZTDSR) – ZTW – ZTG [ZTDSR-ZTW-ZTG]. More details on treatments and management practices followed are given in Table 1. The description of experimental treatments with cropping details is given in Table 1.

**Table 1:** Tillage-*cum*-crop establishments (TCE) drivers for agricultural change of different scenarios.

Treatment notations	Tillage			Crop establishment			Residue management		
	Rice	Wheat	Greengram	Rice	Wheat	Greengram	Rice	Wheat	Greengram
scI: RPTR-BCW-ZTM (FP)	Cultivator: 2 passes (dry tillage: DT) Rotavator: 1 pass (wet tillage: WT)	Cultivator: 2 passes Rotavator: 1 pass	Zero till	25-days old seedlings, manually transplanted with random geometry	Broadcasting	Drill seeding with Happy Seeder	~30% incorporated in soil	~30% retained on soil surface	100% incorporated
scII: LPTR-CTW-ZTM (FP)				25-days old seedlings, manually transplanted in lines at 25 × 15 cm apart.	Drill seeding with Happy Seeder				
scIII: CTMTR-ZTW-ZTM (pCA)		Zero-till		18-days old seedlings, machine transplanting at 23 × 14 cm apart.			~30% retained on soil surface		
scIV: ZTMTR-ZTW-ZTM (CA)	Zero-till (flooding before transplanting)			18-days old seedlings, machine transplanting at 23 × 14 cm apart.					100% removed
scV: SRI-SWI-ZTM (pCA)	Cultivator: 2 passes (DT) Rotavator: 1 pass (WT)	Cultivator: 2 passes Rotavator: 1 pass		12-days old seedlings, manual transplanting at 25 × 25 cm apart.	Manual seeding		~30% incorporated in soil		100% incorporated
scVI: CTDSR-ZTW-ZTM (pCA)	Cultivator: 2 passes Rotavator: 1 pass	Zero-till		Drill seeding at 22.5 cm row spacing	Drill seeding with Happy Seeder		~30% retained on soil surface		
scVII: ZTDSR-ZTW-ZTM (CA)	Zero-till			Drill seeding at 22.5 cm row spacing					100% retained on the soil surface

### 2.2. Soil sampling, earthworms count, and soil parameters analysis

Soil samples were collected from 0 to 15 cm soil depth with soil augur after harvest of wheat in 2022 in aseptic conditions (after 7 years of experimentation). Soils were collected from five randomly selected points in each plot and mixed to get a representative composite sample. One part of the composite sample was used for DNA extraction while other subsample was air-dried and passed through a 2-mm sieve for analysis of physico-chemical properties. Organic carbon (OC), and available N, P, and K content were determined by standard methods (Bray and Kurtz, 1945; Subbiah and Asija, 1956; Jackson, 1973). The micro-nutrient content (Fe, Mn, Zn, and Cu) of the soil was estimated by extraction with diethylene triamine pentaacetic acid (DTPA) followed by atomic absorption spectrophotometer-based quantification using the method of Lindsay and Norvell (1978). Earthworms populations were estimated in standing rice crops during August 2021 and in post-rainy crops during February 2022 by excavating one pit (0.3 m × 0.3 m × 0.3 m) in every plot and hand-sorting method followed (Fonte et al., 2009) to counts per cubic meter. This observation was made early in the morning as earthworms were reported to have higher activity at night (Singh J. et al., 2018).

The soil quality index was created using non-linear techniques (Bastida et al., 2006). Parameters that showed significant differences were used to calculate the minimum dataset (MDS) using principal component analysis (PCA), a multivariate statistical technique (Andrews et al., 2002). The principal components (PCs) with higher eigenvalues (≥1) and factor loadings were considered as the best representation of the system attributes. However, in this study, we only had the first three PCs with eigenvalues greater than 1, which accounted for only 86% of the total variance. To account for more than 90% of the variance, we selected PCs with eigenvalues above 0.9 that explained more than 5% of the total variance for calculating the soil quality index. From each PC, we selected highly weighted factors with absolute values within 10% of the highest factor loading or with eigenvalues greater than 0.40. If multiple factors were retained from a single PC, we considered multivariate correlation analysis, and only factors with correlation coefficients less than 0.60 were included in the MDS. Once we selected all the MDS of soil quality indicators, we generated scores for different parameters using non-linear methods described in the following equation (Bastida et al., 2006). For all MDS indicators, we followed a “more is better” approach except for ECe, where a “less is better” function was used.


$$S=\frac{a}{\left(1+{\left(\frac{x}{x_{0}}\right)}^{b}\right)}$$

Where, ‘ a ’ is the maximum value (1 in our case) reached by the function, ‘x’ is the value of an individual observation of the corresponding parameter, ‘x_0_’ is the mean of all observations for that parameter corresponding to each treatment, ‘b’ is the value of the slope of the equation. The weighted scores of the MDS parameters for each observation were added to get the SQI using followed equation.


$$SQI=\overset{n}{\underset{i=1}{\sum }}W_{i}S_{i}$$

Where, ‘W’ is the weightage of the MDS variable obtained from PCA, ‘S’ is the score of that particular variable.

### 2.3. Genomic DNA extraction and amplicon sequencing

Initial soil processing for DNA extraction was done as per the methodology described by Rincon-Florez et al. (2020). The processed soil samples were further used for DNA extraction using DNeasy Power Soil Isolation Kit (QIAGEN, India) as per instructions of manufacturer. Final DNA concentrations and quality were measured using agarose gel electrophoresis and Qubit fluorometer 4.0 (ThermoFisher Scientific, United States). DNA was PCR-amplified using forward primer (16S_341F: TCG TCG GCA GCG TCA GAT GTG TAT AAG AGA CAG CCT ACG GGN GGC WGC AG) and reverse primer (16S_805R: GTC TCG TGG GCT CGG AGA TGT GTA TAA GAG ACA GGA CTA CHV GGG TAT CTA ATC C) with a KAPA HiFi HotStart Ready Mix (Roche, Basel, CHE) targeting V3-V4 region of 16S rRNA genes. The two-round protocol of PCR was performed as described previously (Naqib et al., 2018). First round of PCR was used as a template without cleanup. To add appropriate sample-specific indices and Illumina flow cell-specific sequences, a second round of PCR was run for 10 cycles. Sequencing libraries were created in accordance with the Illumina 16S library preparation methodology (Illumina, San Diego, CA, USA). Using the Nextera XT Index kit, dual index adapters for sequencing on the Illumina MiSeq platform were connected (Illumina, San Diego, CA, USA). Using the MiSeq Reagent Kit v3, these libraries were sequenced in a 225-bp paired-end run for V3-V4 (600 cycles).

### 2.4. Sequencing data analysis and statistical analysis

After completion of the sequencing run, data from each scenario was analyzed using mothur v.1.30.0 pipeline (Kozich et al., 2013). The quality of all raw sequence reads was assessed according to many criteria, including base call quality distribution, % GC, and sequencing adapter contamination using Fast QC v0.11.8 software and MultiQC tool (Ewels et al., 2016). Raw reads were processed to remove adapter sequences and low-quality bases using TrimGalore V0.4.0 (Krueger et al., 2021). The trimmed reads were further stitched and used into mothur software and aligned with each other to form contigs. Contigs were screened for errors and only those between 300 bp and 500 bp were retained. High-quality contigs were checked for identical sequences and duplicates were merged. As a part of pre-processing of sequence reads, query sequences were clustered using Uclust method (Edgar, 2010) against a curated chimera-free 16S rRNA database using Uchime algorithm implemented in tool Vsearch, version1.7.0 (Rognes et al., 2016). Filtered contigs were processed and classified into taxonomical clusters at ≥97% sequence similarity against reference database based on GreenGenes (GG) v.13.8–99 database (McDonald et al., 2012) which resulted in generation of biom file. Further comparative analyses according to sample wise were performed. Diversity indices *viz.*, Shannon, Simpson, Chao1, Fisher, OTUs (Operational Taxonomic Units), and rarefaction curves were calculated using Mothur. Beta-diversity was analyzed by Principal coordinate analysis (PCoA) based on Bray–Curtis index distance method Analysis of Group Similarities (ANOSIM). With the use of online Microbiome Analyst platform and the Euclidean distance approach, a heatmap based on taxonomic affiliation at the phylum level relative abundance was created (Dhariwal et al., 2017). Using the “VennDiagram” tool in the R environment^1^, a ven diagram was drawn that illustrates the relationships between distinct and shared OTUs across scenarios. Relationship among major bacterial phyla (>1% relative abundance) was established based on correlation analysis and the figure was drawn using online Microbiome Analyst platform with Pearson correlation coefficient algorithm (Chong et al., 2020). The standard error of the mean with respect to each parameter was calculated. To ascertain the effects of various treatments on the diversity of the bacterial population, analysis of the variance (ANOVA) was used. Using SPSS Windows version 21.0, means were separated using Duncan’s multiple range test (DMRT)/Kruskal-Wallis at a 5% level of significance (SPSS Inc., Chicago, IL, United States) (Supplementary Table S1). Data were tested for normality normal distribution before analyses. Principal Components Analysis (PCA) (XLStat 7.5, Addinsoft) was performed to determine the explanatory effects of soil attributes, and earthworms count on bacterial communities. PCA was performed with the dataset of 21 attributes followed by Choudhary et al. (2020). It was considered that the variables in the PCs with eigenvalues >0.7 and those that explained at least 5% of the fluctuation in the data were those that best-represented system features.

## 3. Results

### 3.1. Soil quality parameters and earthworms activity

Comparative evaluation of the soil quality parameters across the different management scenarios revealed that soil properties are significantly influenced by agricultural management. Differences in soil pH among scenarios were non-significant, while CA-based scenarios (scIV and scVII) recorded significantly higher electrical conductivity (EC) than other partial CA and farmer’s practices. Soil organic carbon (SOC) content was highest (9.65 g kg^−1^) in CA-based scenario (scIV) compared to partial CA (scV) and farmers’ practices. Available-N content was significantly higher in CA-based scenario (scIV and scVII) compared to farmer practice (scII), while available-P content was highest (29.72 kg ha^−1^) in CA-based scenario (scVII). Interestingly, both CA & partial CA-scenarios had markedly more available-potassium (K) content than farmers’ practice, however, the highest available-K content (182.2 kg ha^−1^) was recorded in CA-based scenario (scVII). Variation of available iron (Fe) content among all scenarios was found non-significant. However, other micronutrients like manganese (Mn), zinc (Zn), and copper (Cu) showed higher content of 38.6, 10.9, and 9.9 mg kg^−1^, respectively in CA-scenario (scVII) (Table 2).

**Table 2:** Earthworm counts and soil chemical properties in different scenarios of agricultural management.

Scenario	pH	EC (dS m^−1^)	SOC (g kg^−1^)	N (kg ha^−1^)	P (kg ha^−1^)	K (kg ha^−1^)	Fe (mg kg^−1^)	Mn (mg kg^−1^)	Zn (mg kg^−1^)	Cu (mg kg^−1^)	Earthworm counts (no. m^−3^)
scI	7.34 ± 0.06^a^	0.12 ± 0.01^b^	8.62 ± 0.24^c^	212.9 ± 8.39^ab^	23.5 ± 0.61^c^	128.5 ± 9.06^d^	155.6 ± 3.26^a^	31.1 ± 0.87^bc^	10.40 ± 0.35^a^	7.10 ± 0.29^c^	343 ± 5.3^e^
scII	7.42 ± 0.08^a^	0.10 ± 0.00^b^	8.81 ± 0.17^bc^	204.3 ± 7.17^b^	27.2 ± 0.90^ab^	129.8 ± 2.83^d^	142.1 ± 4.92^a^	29.3 ± 0.58^c^	8.27 ± 0.33^c^	6.82 ± 0.20^c^	625 ± 9.9^d^
scIII	7.24 ± 0.05^a^	0.13 ± 0.01^b^	9.45 ± 0.02^ab^	227.4 ± 3.32^ab^	26.0 ± 0.13^bc^	144.1 ± 0.7^cd^	156.1 ± 3.10^a^	38.3 ± 0.37^a^	9.40 ± 0.42^abc^	8.03 ± 0.61^bc^	766 ± 19.8^c^
scIV	7.34 ± 0.06^a^	0.11 ± 0.01^b^	8.90 ± 0.08^bc^	237.1 ± 13.08^ab^	28.0 ± 0.82^ab^	169.4 ± 3.88^ab^	156.4 ± 1.71^a^	31.0 ± 0.69^bc^	9.80 ± 0.23^abc^	9.63 ± 0.20^a^	1,024 ± 25.4^b^
scV	7.49 ± 0.05^a^	0.25 ± 0.02^a^	9.65 ± 0.13^a^	243.8 ± 4.22^a^	29.1 ± 1.00^ab^	156.4 ± 4.35^bc^	147.1 ± 4.75^a^	35.2 ± 2.46^ab^	9.93 ± 0.44^ab^	9.20 ± 0.26^ab^	1,130 ± 14.5 ^a^
scVI	7.13 ± 0.03^a^	0.15 ± 0.02^b^	9.05 ± 0.16^abc^	236.5 ± 3.97^ab^	28.7 ± 0.96^ab^	161.9 ± 5.03^abc^	157.7 ± 2.30^a^	36.5 ± 1.53^a^	8.53 ± 0.29^bc^	9.21 ± 0.12^ab^	802 ± 9.2^c^
scVII	7.24 ± 0.07^a^	0.30 ± 0.04^a^	9.36 ± 0.08^ab^	247.4 ± 7.33^a^	29.7 ± 0.46^a^	182.2 ± 6.73^a^	155.6 ± 2.80^a^	38.7 ± 0.58^a^	10.87 ± 0.35^a^	9.90 ± 0.15^a^	660 ± 72.7 ^d^

The same superscript letters of means values within each column are not statistically different (Duncan’s multiple range test; *p* ≤ 0.05). Values are the average of five replicates (*n* = 5); (means ± mean of standard error). pH, Potential of Hydrogen, EC, electrical conductivity; SOC, soil organic carbon; N, P, K, available nitrogen, phosphorus, and potassium; Cu, Zn, Fe, Mn, DTPA extractable copper, zinc, iron, and manganese.

**Figure 1:**
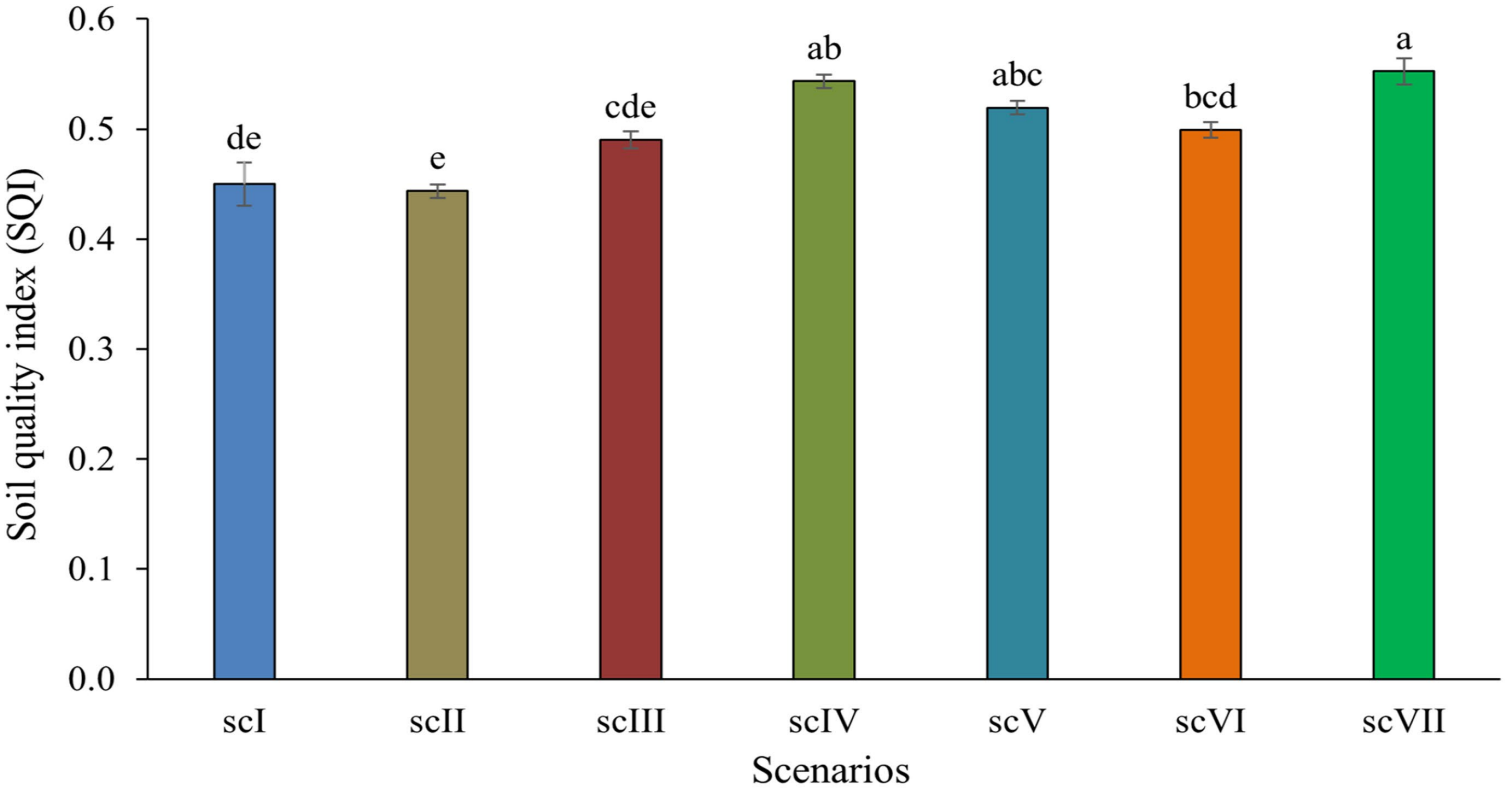
Soil quality index (SQI) as affected by different tillage-*cum*-crop establishments scenarios (sc). Vertical bars represent standard error of mean. Means followed by different small letters are significantly different at *p* ≤ 0.05.

Earthworm counts across different management scenarios revealed that CA-based scenario (scIV) had statistically higher (1,024 ± 25.4 no. m^−3^) earthworms compared to other management scenarios. Partial (scIII, scV, and scVI) and full CA-based management scenarios had comparatively higher numbers of earthworm counts over farmers’ practices (scI and scII). The lowest number of earthworms count (343 ± 5.3 no. m^−3^) was observed in conventional farmers’ practices (scI) (Table 2). Soil quality index differed significantly among various treatments. The highest SQI of 0.55 was noted in scVII (0.55) and it was significantly higher than scI, scII, scIII and scVI but was at par with scIV and scV (Figure 1).

### 3.2. Diversity of bacterial community

Bacterial community profiling from extracted DNA of 35 soil samples (7 treatments each replicated five times) yielded a total of 8,209,304 raw reads obtained for V3-V4 regions. After quality filtering of sequences, 2,328,785 reads were processed and used for identification purposes. Final sequence of all scenarios had submitted to the NCBI with Bio-project: PRJNA914075. The asymptotic nature of the rarefaction curve was achieved at reading depths of 60,000 sample size indicating saturation of bacterial communities profiling (Supplementary Figure S1). Significantly higher number of OTUs (1,192 ± 50) and Chao1 (1415.65 ± 14.34) values were observed in scIII (pCA) (*p* ≤ 0.05) (Table 3). Shannon diversity index showed that there was a 2–3% variation among different scenarios. Diversity indices values of Simpson, ace, and fisher were higher under CA and pCA (scIV, scIII scenarios) compared to the FP-based agricultural management scenarios (scI, scII scenario) (Table 3).

**Table 3:** Diversity indices (mean ± standard error of mean) of bacterial communities of seven scenarios for clusters at >97% sequence similarity cut-off against reference database.

Scenario*	Shannon	Simpson	Chao1	Ace	Fischer	OTU
scI (FP)	5.07 ± 0.03^b^	0.983 ± 0.001^bc^	1213.01 ± 14.50^ab^	1199.94 ± 16.72^ab^	122.52 ± 3.53^ab^	1,040 ± 34^a^
scII (FP)	4.97 ± 0.04^ab^	0.982 ± 0.003^ab^	1123.38 ± 12.59^a^	1120.66 ± 13.52^a^	114.20 ± 2.43^a^	970 ± 36^a^
scIII (pCA)	5.07 ± 0.04^b^	0.982 ± 0.001^ab^	1415.65 ± 14.34^b^	1389.13 ± 13.84^b^	129.43 ± 2.97^b^	1,192 ± 50^b^
scIV (CA)	4.94 ± 0.01^a^	0.980 ± 0.000^a^	1223.34 ± 17.92^ab^	1198.22 ± 16.13^a^	120.18 ± 2.76^a^	1,028 ± 30^a^
scV (pCA)	5.05 ± 0.04^b^	0.984 ± 0.002^c^	1198.27 ± 18.30^a^	1199.10 ± 19.31^a^	121.91 ± 3.88^ab^	1,054 ± 25^a^
scVI (pCA)	4.98 ± 0.01^ab^	0.982 ± 0.001^ab^	1193.28 ± 16.10^a^	1179.56 ± 15.98^a^	119.21 ± 2.75^a^	1,013 ± 43^a^
scVII (CA)	5.02 ± 0.03^ab^	0.984 ± 0.001^bc^	1228.85 ± 07.50^ab^	1192.79 ± 66.80^a^	116.26 ± 0.90^a^	999 ± 19^a^

OTU, Operational taxonomic unit; Same superscript letters of means values within each column are not statistically different (Duncan’s multiple range test; *p* ≤ 0.05).

**Figure 2:**
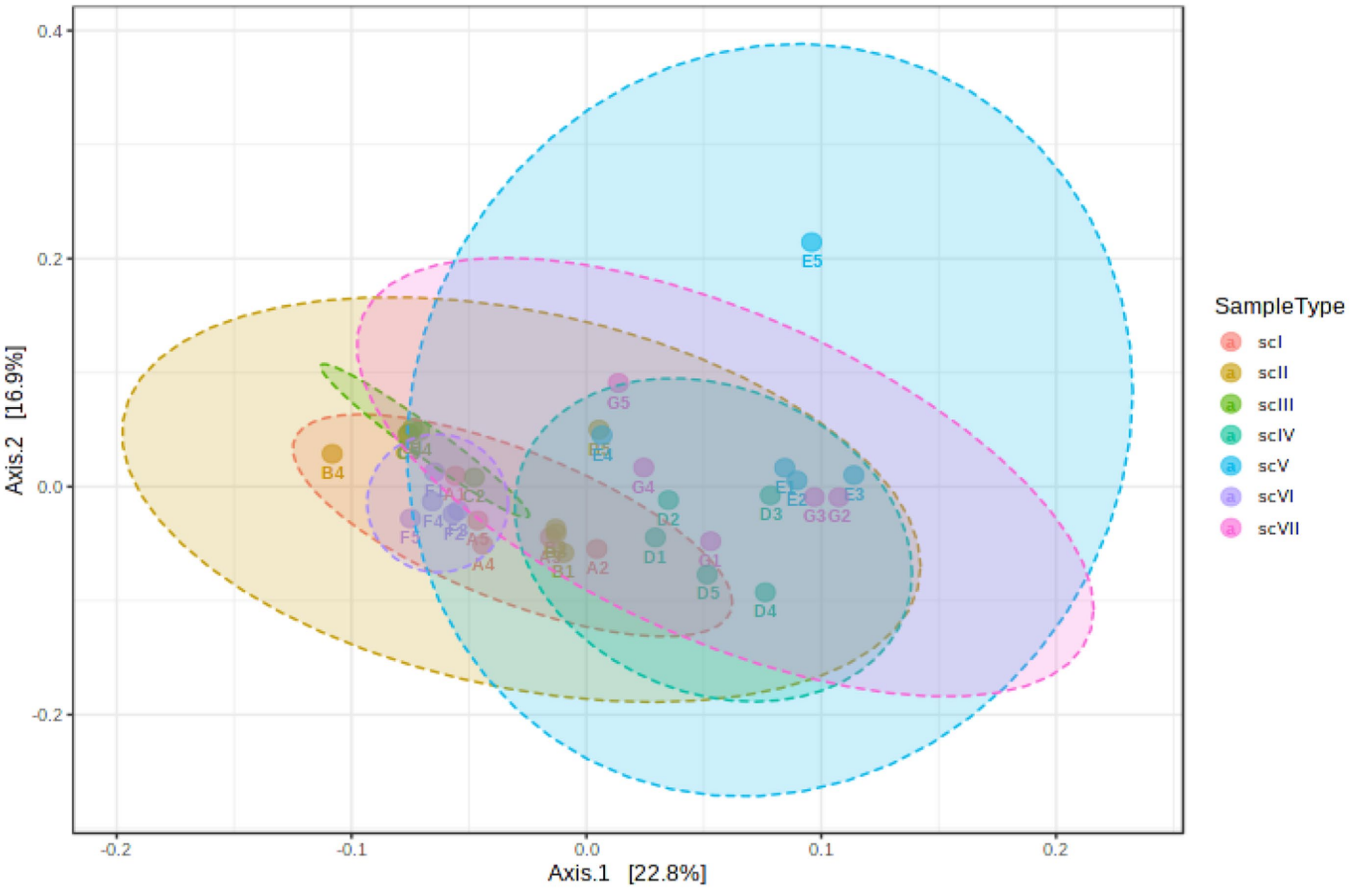
Principal coordinate analysis (PCoA) based on the Bray–Curtis dissimilarity matrix showing significantly different microbial compositions among different scenarios (ANOSIM *R* = 0.61818, value of *p* < 0.001).

Principal coordinate analysis (PCoA) of soil bacterial beta-diversity was performed based on Bray–Curtis index distance method to determine significant differences between various scenarios (Figure 2). Analysis indicated that significant differences among treatments (*R*-value:0.61818; *p-*value≤0.001), confirmed marked impacts of management scenarios on microbial communities compositions. Scenario ScIV had highest difference in taxonomical richness followed by ScV and ScVII scenarios. Comparatively less difference was observed in scII, scIII, and scI scenarios.

### 3.3. Community structures of bacteria

**Figure 3:**
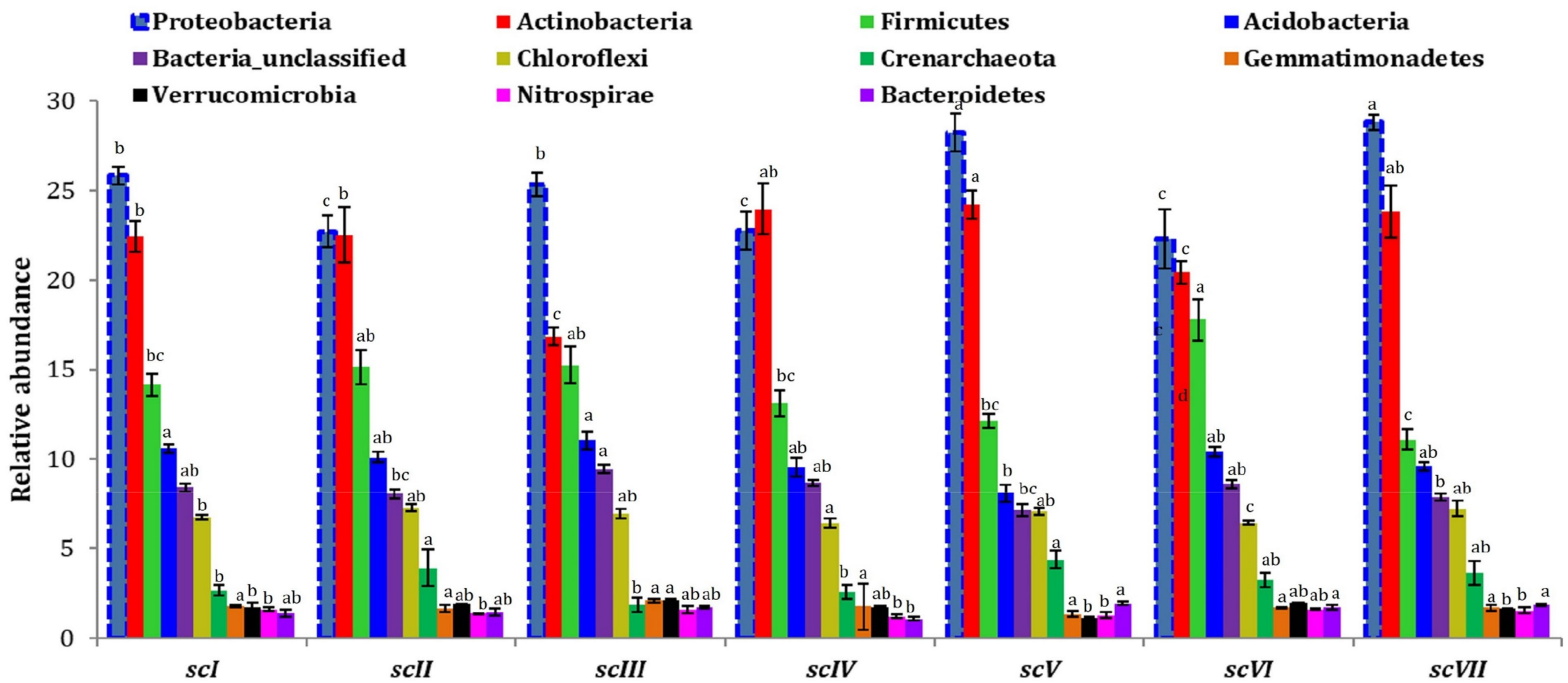
Distribution of the dominating bacterial phyla (>1% relative abundance) in different tillage-*cum*-crop establishments scenarios. The values are average of the five replicates (*n* = 5); whiskers represent standard error of mean (SEm); same letters over bars across different scenarios indicates non-significant differences within phyla (Duncan’s multiple range test; *p* ≤ 0.05).

**Figure 4:**
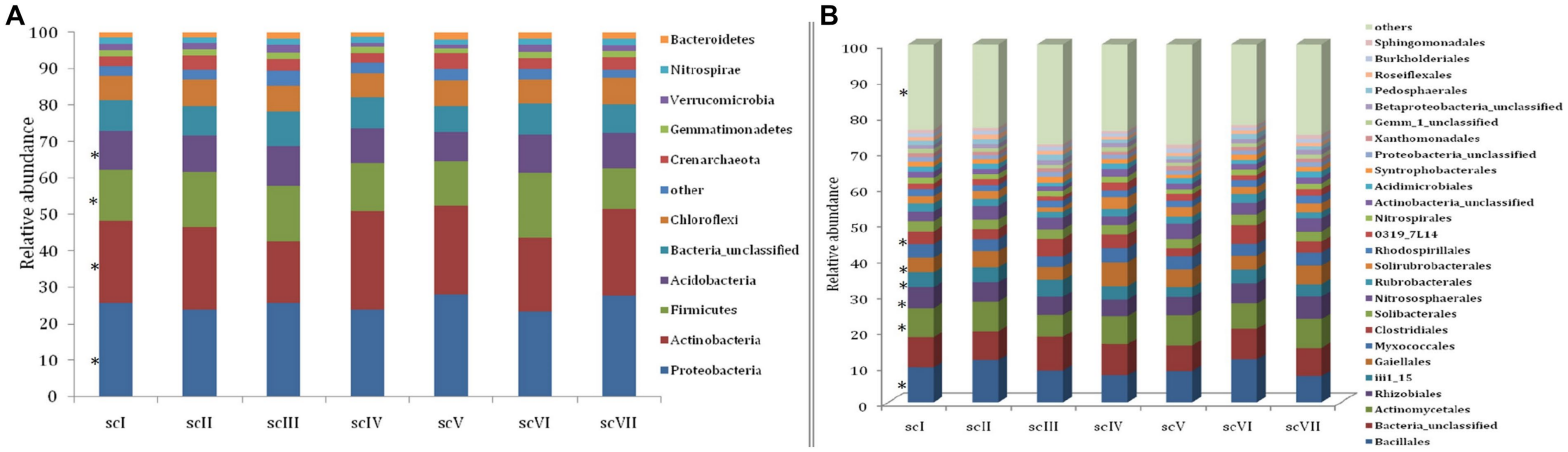
Relative abundance at phylum, Order, and OTU level in different tillage-*cum*-crop establishments. **(A)** Relative abundance of dominant bacterial phyla **(B)** Relative abundance of dominant bacterial orders. Taxa below 1% relative abundance were clubbed together as “others.” Taxa with asterisk (*) mark are statistically differed among treatments (scenarios) (Kruskal–Wallis test; *p* ≤ 0.05).

Analysis of operational taxonomic units (OTUs) of bacterial community detected 42 phyla in seven scenario. Changes in number and composition of bacterial communities across the scenarios indicate that selection of crops, tillage, and residue management play important roles in the community structure of bacteria (Figure 3). Top five phyla namely *Proteobacteria, Actinobacteria, Firmicutes, Acidobacteria,* and *Chloroflexi* with an abundance of more than 5% were represented by~88% of total phyla in each scenario (Figure 4A). Core bacterial phyla in all scenarios were mainly *Proteobacteria* (ranging from 23.30% in scVI to 28.3% in scV scenario), *Actinobacteria* (ranging from 16.8% in scIII to 24.2% in scV scenario), and *Firmicutes* (ranging from 11.1% in scVIII to 17.78% in scVI scenario) (Figure 3). Comparative abundance of *Proteobacteria* had 2–13% more under pCA and CA-scenario (scIII, scIV, scVI, scV, and scVII) over the farmers’ practices (scI and scII). *Actinobacteria* was comparatively more under CA (scIV, and scVII) than pCA and farmers’ practices-based scenarios. The abundance of *Firmicutes* was significantly higher (15–23%) higher in farmers’ practices and partial CA-based scenarios (scI, scII, scIII, scV, scVI) over CA-based scenarios. Similarly, the comparative richness of *Acidobacteria* and *Chloroflexi* were markedly (*p* ≤ 0.05) better in the case of farmers’ practices and partial CA over CA-based scenarios. However, abundance of *Crenarchaeota* was found less than 5% (~3.0%) among bacteria phyla but comparative richness were statistically more under CA-based scenario (scIV, and scVII) over others. *Bacteroidetes* phylum was more under CA- scenarios than FP. Clustering patterns and comparative richness of bacterial phyla contributes>1% across seven scenarios were depicted through a clustering heatmap (Supplementary Figure S2). Heatmap clustering of OTUs from diverse scenarios revealed that different bacterial communities were associated with different agricultural management practices. Presence of major phyla, i.e., *Proteobacteria, Actinobacteria, Firmicutes*, etc. associated differently with either CA or other farmers’ based practices.

**Figure 5:**
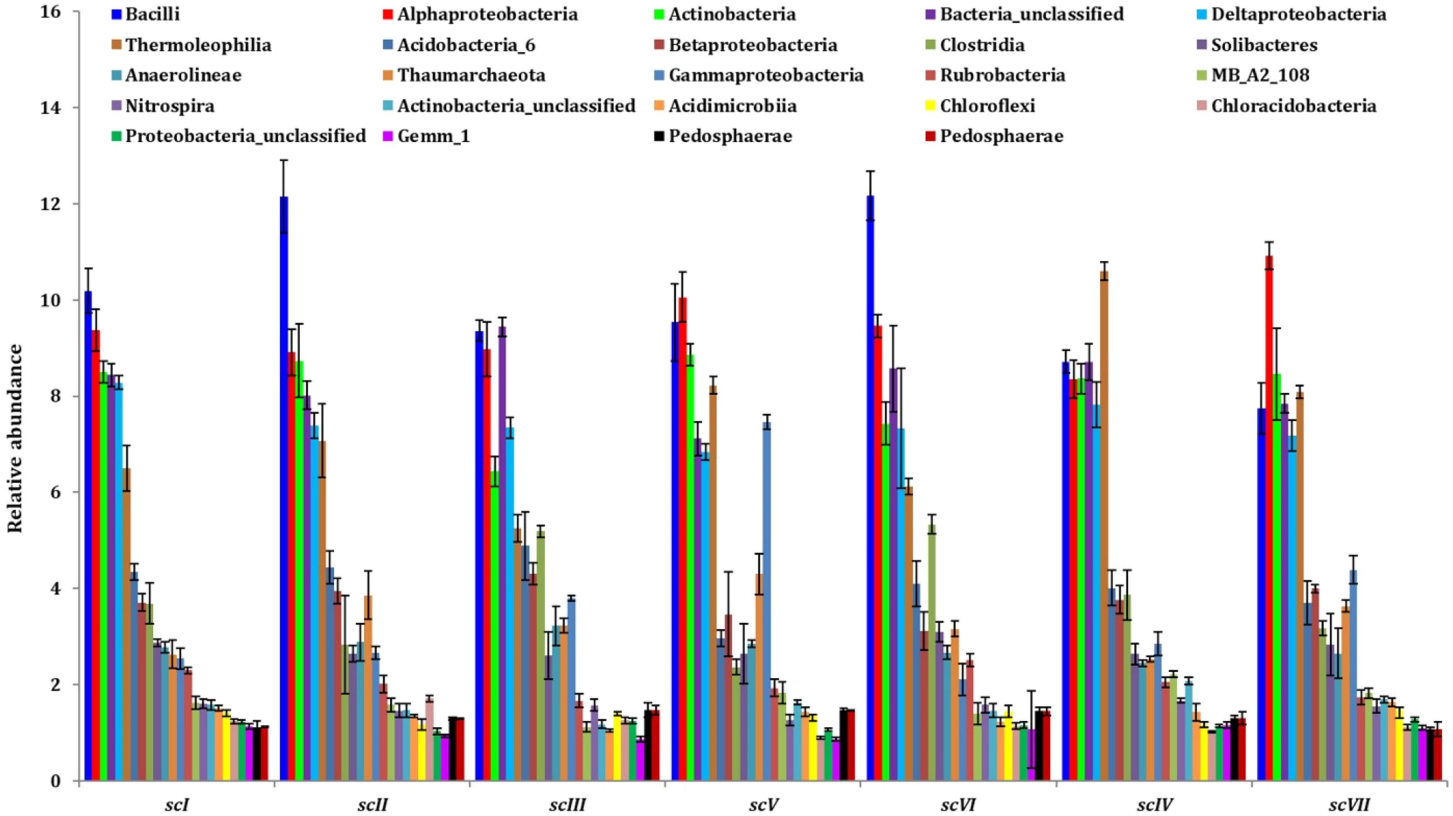
Distribution of dominating classes (>1% relative abundance) in different tillage-*cum*-crop establishments scenarios. The values are average of five replicates (*n* = 5); whiskers represent standard error of mean (SEm).

**Figure 6:**
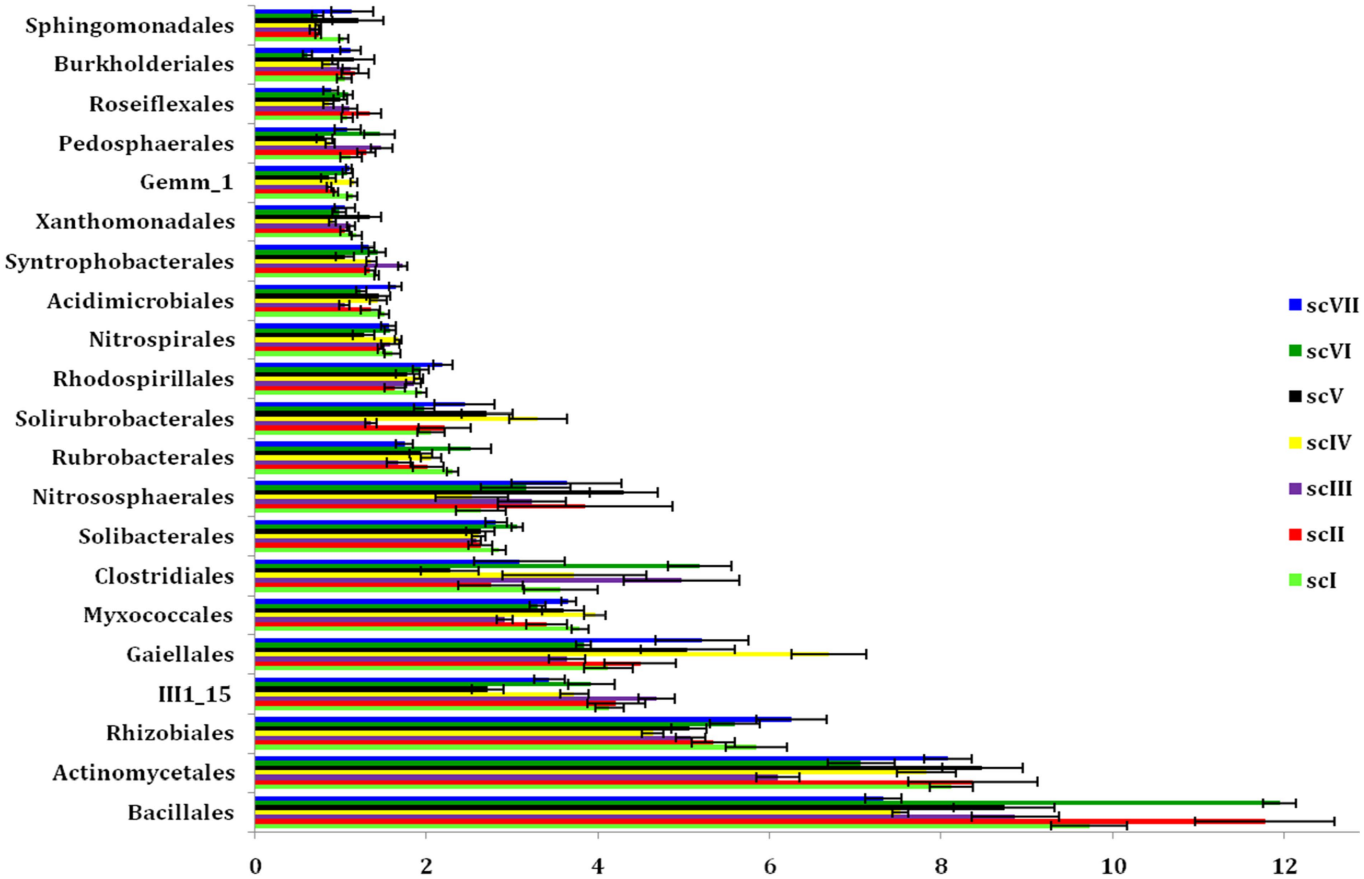
Dominating orders (>1% relative abundance) under the different tillage-*cum*-crop establishments scenarios. The values are average of the five replicates (*n* = 5); whiskers represent standard error of the mean (SEm).

Bar diagram revealed higher differences in relative abundance of classes in seven scenarios of agriculture management (Figure 5). Class *Bacilli* of phylum *Firmicutes* were the most abundant followed by *Alphaproteobacteria* (phylum *Proteobacteria*) and *Actinobacteria* (phylum *Actinobacteria*) under all scenarios except scIII and scIV, where unclassified bacteria were abundant. Class *Bacilli* was highest in scVI (12.2 ± 0.51%) followed by scII (12.1 ± 0.75%). Abundance of *Alphaproteobacteria* was highest in CA based scenarios (10.1 ± 0.23% and 10.9 ± 0.27% in scV and scVII, respectively) followed by pCA and farmers’ practices. Most dominant phylum *Proteobacteria*, comprising, i.e., *Alphaproteobacteria, Deltaproteobacteria, Betaproteobacteria,* and *Gammaproteobacteria* represented 25% of sequences in different scenario. Further, relative abundance analysis of these classes was performed at order level (Figures 4B, 6). Average relative abundance of order *Bacillales* was the highest (9.41%) followed by *Actinomycetales* (7.71%) and *Rhizobiales* in all the scenarios. Relative abundance of *Bacillales* was 9–21% higher in farmers’ practices-and partial CA- scenario (scI, scII, scIII, scV, and scVI) compared to CA-based scenarios. Order *Actinomycetales* was relatively higher in CA-based scenarios (scIV and scVII) over to pCA and farmers’ practices. Relative abundance of *Rhizobiales* was significantly high in CA-based scenarios (scIV and scVII) compared to pCA and farmers’ practices-based scenarios. Relative abundances of order *Bacillales, Actinomycetales, Rhizobiales, Gaiellales,* and *Nitrososphaerales* were significantly different among seven scenarios of agriculture management (Figure 4B).

### 3.4. Interactions of bacterial community and soil chemical properties

**Figure 7:**
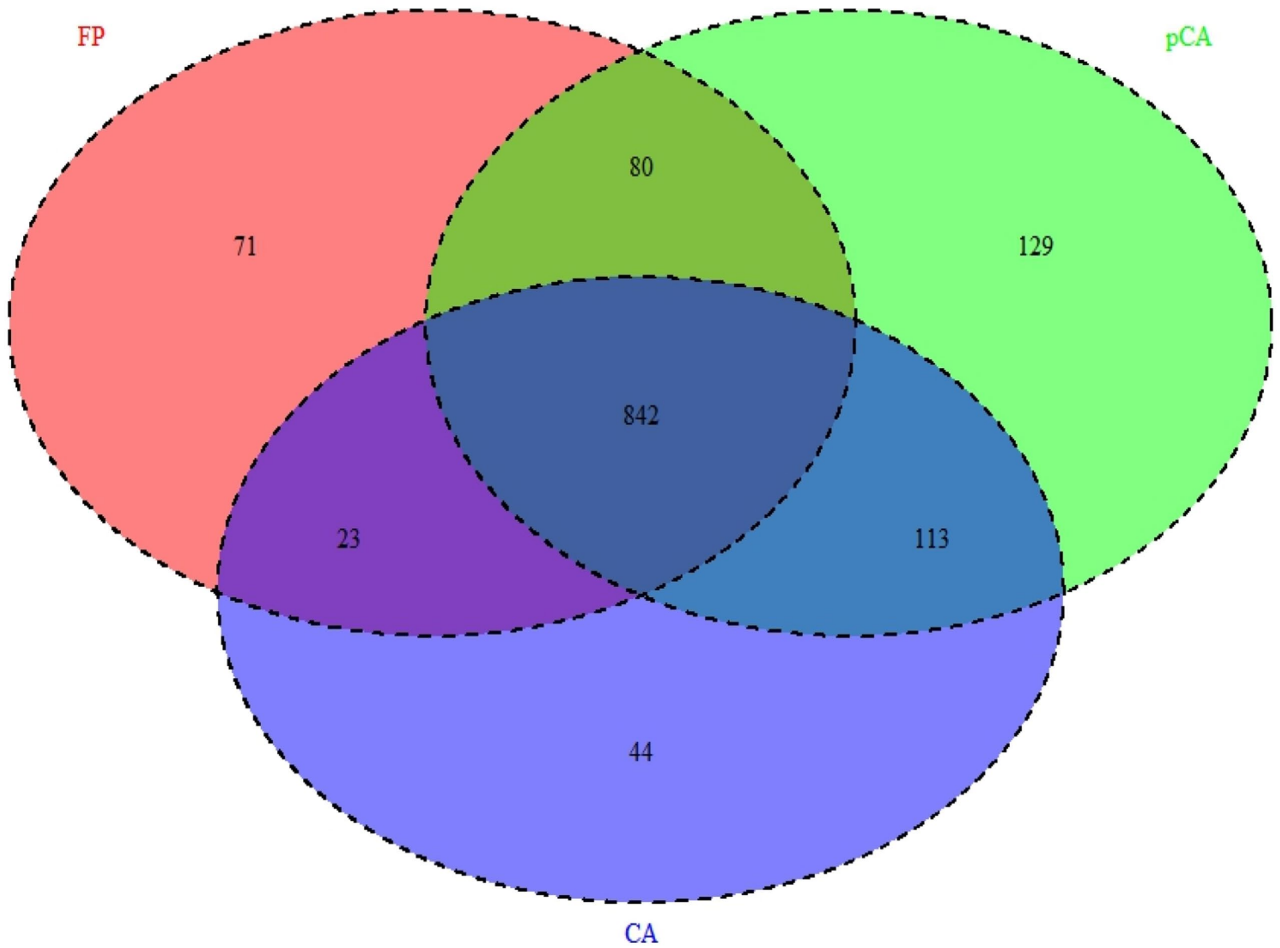
Venn diagram showing unique and shared OTUs between different tillage- *cum*- crop establishment and residue management production systems.

**Figure 8:**
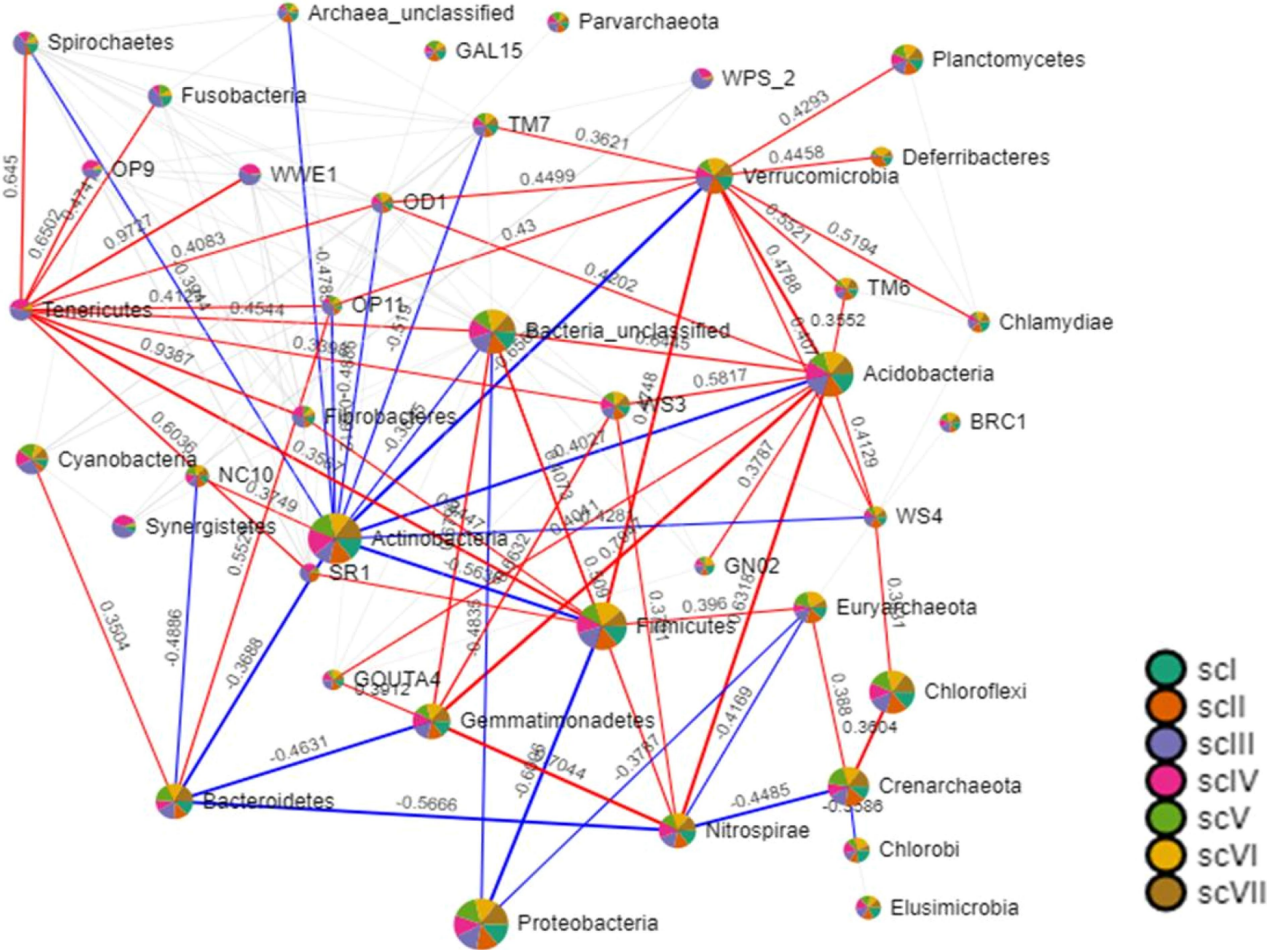
Relationship among the major bacterial phyla (>1% relative abundance) of tillage-*cum* crop establishment and residue management production system scenarios. Size of nodes is based on number of connections to that node (phylum). Two phyla are connected through bars which size is based on magnitude of correlation. Blue color of bars represents negative correlations, while red represents the positive correlations.

It appears that CA principles had positive impacts on bacterial community compositions and structures. These differences reflect turnovers, such as replacement of one species/taxon by another, or assemblage to one another (nestedness) (Baselga, 2010). Relationship between OTUs presence in different crop management, i.e., farmers’ practices, partial CA, and full CA has been depicted through Venn diagram, which revealed that 842 OTUs shared among all crop management scenarios at 97% similarity cut-off (Figure 7). Relationship networks among the phyla showed a positive or negative relationship based on tillage-*cum*-crop-establishment and residue management production system scenarios among the major bacterial phyla (Figure 8). The presence of *Proteobacteria* negatively correlated with *Firmicutes,* where their composition differed among scenarios as shown above. The observed relationship among phyla is due to the occurrence of specific phyla, that are sensitive to soil conditions produced by different crop management practices.

**Figure 9:**
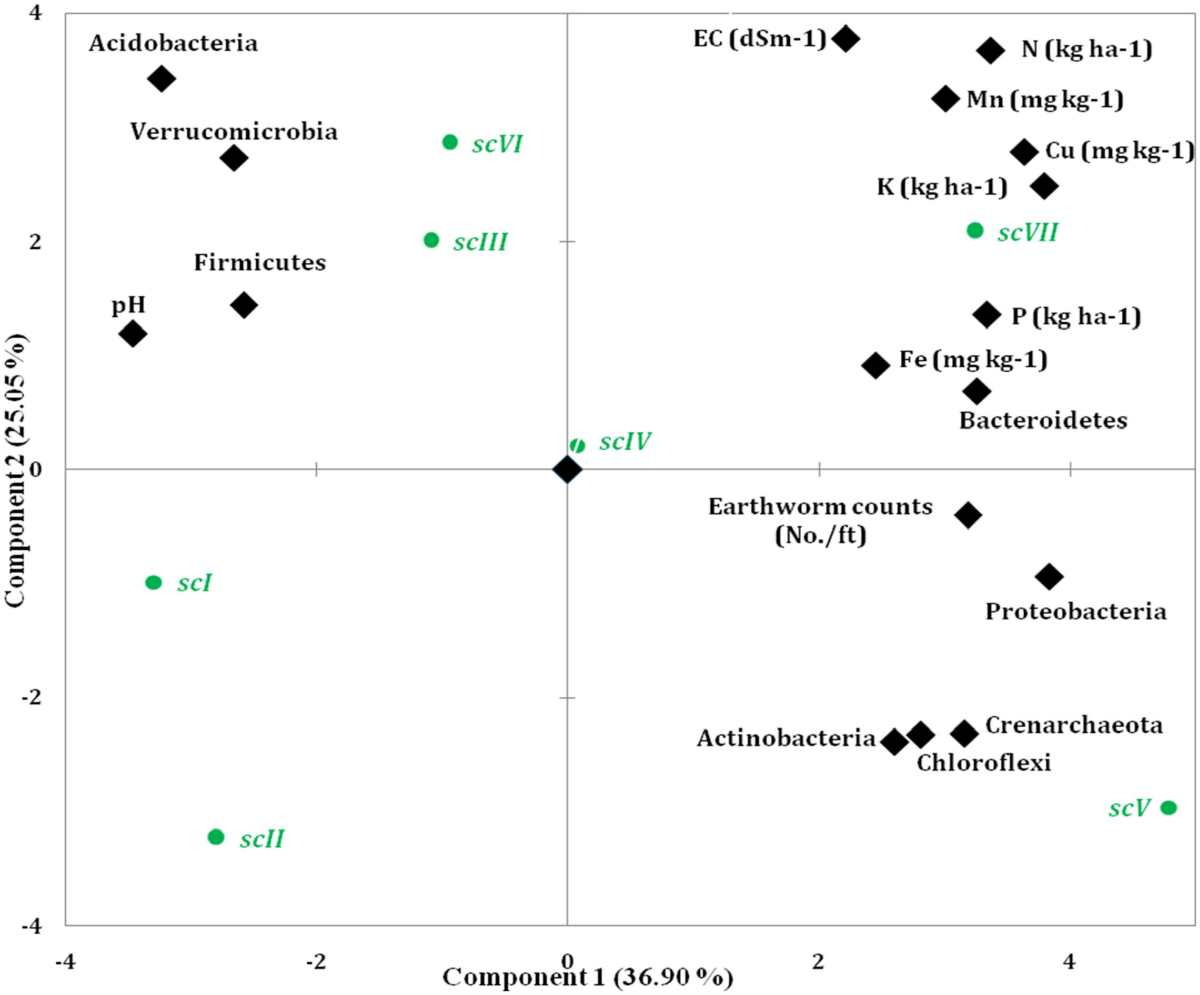
Scatter plot of seven scenarios of agricultural managements on PCA coordinates based on major bacterial phyla, earthworm counts and different soil properties.

Principal Component Analysis (PCA) showed that soil variables and earthworm population significantly affected bacterial community composition and structures (Figure 9 and Table 4). PCA was generated using different soil properties, earthworm count, and top 10 bacterial phyla. Eigenvalues with >0.9 which represent 91.44% of total variance were extracted into four principal components. Total addressed variance (91.44%) was decomposed in PC1 for 36.9% and PC2 for 25.05%, whereas PC3 and PC4 accounted, for 17.52 and 11.98%, respectively of total variance (Figure 9 and Table 4). *Proteobacteria*, K, Cu, P, N, *Acidobacteria*, *Crenarchaeota*, and *Bacteroidetes* were with higher loading in PC1. Based on component loading, SOC, N, P, Fe, Cu, *Proteobacteria, Gemmatimonadetes*, and *Nitrospirae* were most important parameters among 21 variables as affected by TCE and residue management scenarios.

**Table 4:** Principal components (PC) and factor loadings extracted from major bacterial phyla, earthworm counts and different soil properties; bold loading extracts were used to explain PC.

Principal components	PC1	PC2	PC3	PC4
Eigenvalue	7.75	5.26	3.68	2.52
Variability (%)	36.90	25.05	17.52	11.98
Cumulative %	36.90	61.94	79.46	91.44
Factor loading/Eigen vector				
pH	−0.731	0.208	0.177	−0.594
EC (dSm^–1^)	0.467	0.657	−0.091	−0.208
SOC (g kg^–1^)	0.305	**0.778**	−0.427	−0.330
N (kg ha^–1^)	**0.712**	0.640	0.185	−0.162
P (kg ha^–1^)	**0.705**	0.236	0.052	−0.635
K (kg ha^–1^)	**0.801**	0.432	0.362	−0.134
Fe (mg kg^–1^)	0.518	0.158	0.245	**0.746**
Mn (mg kg^–1^)	0.637	0.566	−0.471	0.225
Zn (mg kg^–1^)	0.001	**0.707**	0.387	0.364
Cu (mg kg^–1^)	**0.768**	0.486	0.362	−0.187
Earthworms	**0.672**	−0.069	0.125	−0.348
*Proteobacteria*	**0.809**	−0.163	−0.336	0.432
*Actinobacteria*	0.549	−0.415	**0.701**	−0.155
*Firmicutes*	−0.545	0.251	−0.426	−0.480
*Acidobacteria*	**−0.683**	0.597	−0.354	0.192
*Chloroflexi*	0.593	−0.404	−0.619	0.063
*Crenarchaeota*	**0.668**	−0.402	−0.582	−0.208
*Bacteroidetes*	**0.689**	0.119	−0.647	0.165
*Verrucomicrobia*	−0.560	0.476	−0.671	−0.063
*Gemmatimonadetes*	−0.398	**0.800**	−0.044	0.306
*Nitrospira*	−0.273	**0.787**	0.454	0.140

Similarly to prevent redundancy of PCA, Pearson’s correlation was done among bacterial phyla (10 no.), soil properties and earthworm count (Table 5). pH was significantly negatively correlated with *Proteobacteria* (*r* = −0.93, *p* < 0.001), *Bacteroidetes* (*r* = −0.68, *p* < 0.05), and *Chloroflexi* (*r* = 0.66, *p* < 0.05) whereas, positively associated with *Firmicutes* (*r* = 0.65, *p* < 0.05). EC, SOC, N, P, K, and Fe did not have a significant correlation with bacterial phyla. The presence of Mn was strongly interrelated with phylum *Bacteroidetes* (*r* = 0.84, *p* < 0.05), while Zn with phylum *Nitrospirae* (*r* = 0.65, *p* < 0.05). Earthworm count was positively correlated with the presence of Cu in different scenarios.

**Table 5:** Relationship between soil properties, earthworms and major bacterial phyla in different agriculture management scenarios.

Bacterial phyla	pH	EC (dSm^−1^)	SOC (g kg^−1^)	N (kg ha^−1^)	P (kg ha^−1^)	K (kg ha^−1^)	Fe (mg kg^−1^)	Mn (mg kg^−1^)	Zn (mg kg^−1^)	Cu (mg kg^−1^)
Earthworms	−0.38	−0.02	0.28	0.54	0.54	0.57	0.27	0.26	−0.41	0.65*
Proteobacteria	−0.93**	0.22	0.12	0.36	0.36	0.38	0.65	0.67*	−0.07	0.34
Actinobacteria	−0.25	−0.02	−0.41	0.29	0.29	0.52	0.28	−0.25	−0.04	0.50
Firmicutes	0.65*	−0.11	0.40	−0.16	−0.16	−0.48	−0.57	−0.12	−0.26	−0.32
Acidobacteria	0.43	0.00	0.36	−0.20	−0.20	−0.44	−0.18	0.12	0.30	−0.39
Chloroflexi	−0.66*	0.14	0.09	−0.01	−0.01	0.11	0.05	0.46	−0.46	0.00
Crenarchaeota	−0.54	0.19	0.20	0.13	0.13	0.19	−0.05	0.43	−0.55	0.14
Bacteroidetes	−0.68*	0.38	0.54	0.46	0.46	0.31	0.40	0.84*	−0.12	0.34
Verrucomicrobia	0.43	0.16	0.50	−0.22	−0.22	−0.47	−0.45	0.21	0.08	−0.44
Gemmatimonadetes	0.21	0.19	0.43	0.14	0.14	0.01	0.13	0.30	0.56	0.01
Nitrospirae	0.31	0.17	0.32	0.37	0.37	0.27	0.25	0.10	0.65*	0.33

*Significance at the *p* < 0.05; **Significance at the *p* < 0.001.

## 4. Discussion

The present study was focussed on the impacts of long term tillage-*cum*-crop establishment and residue management practices on the bacterial diversity and community composition under the sub-tropical humid climate of Eastern IGP. Soil-bacterial communities have been reported to be greatly affected by the tillage (Hartman et al., 2018). Some researchers have reported that tillage enhances bacterial diversity (Lienhard et al., 2014; Degrune et al., 2016), while others have reported negative impacts (Ceja-Navarro et al., 2010; Constancias et al., 2014). In the present investigation, tillage and crops residue incorporation resulted in more bacterial diversity in partial CA-based scenarios (scIII and scVI) compared to complete CA-scenario. Lower diversity in CA-based scenarios may be due to lower contact of crop residue with soil which is resulting in a equilibrium among different phyla as also reported by Tyler (2019). High bacterial diversity in tillage scenarios may be due to disruption caused by ploughing which releases organic matter and ultimately augments bacterial proliferation (Paustian et al., 2000; Chandra et al., 2023). Crop diversification also creates the differences in bacterial diversity among scenarios (Choudhary et al., 2020). Divsersified cropping system have the different roots exudates that easily hold available compounds, such as amino acids and carbohydrates, which can also stimulate the bacterial population and their composition (Rai et al., 2021). Plant-root association with bacteria is affected by type and use of exudates secreted by roots of different plants (Chandra et al., 2022). Beta diversity analysis showed that scenario scIV had higher differences in taxonomical richness followed by scV, and scVII scenarios. Comparatively lower diversity was observed in scII, scIII, and scI scenarios. The lower level classification yielded diverse or inconsistent results, which may be attributed to a variety of factors including diverse TCE and residue management that directly or indirectly affect composition of microbial population (Venter et al., 2016; Babin et al., 2019). The CA-based practices enhance physico-chemical characteristics of the soil and availability of nutrients, which may also account for various bacterial group distributions observed in various crop settings (Choudhary et al., 2020).

The present study showed that *Proteobacteria* was dominating phyla with high relative abundance in pCA and CA-scenarios. The relative abundance of *Firmicutes, Acidobacteria,* and *Chloroflexi* were noted higher in farmers’ practices and partial *CA.* Crop management directly affected bacterial community composition and indirectly lead to changes in soil environment (Dong et al., 2017; Choudhary et al., 2020). The *Proteobacteria, Acidobacteria, Actinobacteria, Firmicutes,* and *Chloroflexi* were identified as dominant phyla in different crop management practices by other (Degrune et al., 2016; Chen et al., 2018; Tyler, 2019; Choudhary et al., 2020). The most dominating bacterial phylum *Proteobacteria* is copiotrophics in nature, that means it flourishes well under condition of higher carbon availability (Ho et al., 2017). The reports suggest that *Proteobacteria* and *Firmicutes* were the most abundant under less disturbed soil, while *Acidobacteria* and *Chloroflexi* are in farmers practices due to oligotrophics nature lifestyles which flourish well in low nutrition condition (Fierer et al., 2012; Koyama et al., 2014; Ho et al., 2017; Legrand et al., 2018). Ratio of copiotrophic (*Proteobacteria*) and oligotrophic (*Acidobacteria* and *Cholroflexi*) provide intuition into the soil nutritional availability (Choudhary et al., 2020). Higher ratios and nutrient status in CA-based scenarios (scIV, and scVII) are indicators of high copiotrophics and a low number of oligotrophic bacterial phyla in soil. Higher enzymatic activities in CA-based agricultural practices also accelerate mineralization of organic matter, which leads to high availability of nutrient to microbes, ultimately favours copiotrophic bacteria (Jat et al., 2020). Soil analyses revealed that SOC, N, P, K, and Zn along with total earthworms count were found to be significantly higher in CA-based scenarios compared to farmers’ practices, which were also confirmed by Patra et al. (2019) and Choudhary et al. (2020). A high abundance of *Firmicutes* was positively correlated with high phosphate levels that have accumulated from external inputs of inorganic and organic fertilizers (van Bruchem et al., 1999). *Firmicutes*, however, had been noted as dominant amongst the bacterial community in undisturbed forest soil of Kashmir, India (Ahmad et al., 2009), and in ornithogenic soils in Ross Sea region of Antarctica (Aislabie et al., 2012). Hence, the relative abundance of *Firmicutes* was higher under undisturbed no-till CA-based scenarios than in tillage-based farmers practices. Higher abundance of *Chloroflexi* in highly disturbed soil and also under absence of residues cover based scenarios of farmer’s practices were noted and plays a role in cellulose degradations and carbon-cycling (Cole et al., 2013; Pepe-Ranney et al., 2016). Member of this phylum contains varied phenotype with wide-ranging metabolic lifestyle, i.e., photoautotroph, aerobic thermophile, and anaerobic halorespire (Hug et al., 2013).

A higher abundance of *Bacilli,* one of the predominant bacterial classes found in soil, was noted in scII, scVI and scI scenarios. Similar findings have also been reported for the diverse ecological niches (Saxena et al., 2020). *Bacilli* class of bacteria serve many ecological function in the soil ecosystem due to its tremendous genetic and metabolic diversity (Saxena et al., 2020). Several direct and indirect mechanism of plant growth promoting functions by order *Bacialles* are reported by order *Bacillus* (Goswami et al., 2016). More richness of class *Alphaproteobacteria* in CA-scenario (scIV and scVII) can be recognized by congenial environment created in a long-term experiment that favoured *Alphaproteobacteria* (Choudhary et al., 2020). On the other hand, more richness of *Acidobacteria* in farmers’ practices was due to lower status of nutrient found in these scenarios (Legrand et al., 2018). *Rhizobiales*, a recognized order which consists of member of atmospheric N-fixer, are symbiotic and was preferred for CA-based scenarios (Jiménez-Bueno et al., 2016; Kerdraon et al., 2019). Different pattern of bacterial diversities in same soil in similar condition can be attributed to a wide range of factors (Quince et al., 2017). It might be due to sampling timing/sample size in wheat crop. Although contributions of a specific phylum/class to agro-ecosystems is abundant, meager is noted how microbial diversity affect ability of a system to function (van der Heijden and Wagg, 2013). Despite existence of numerous genera/species under various management system, little is known about their possible ecological role in soil.

Earthworm population under CA-based scenarios was statistically at par with partial-CA system but markedly higher as compared to other production scenarios. Undisturbed zero tillage-based practices showed the strong association with earthworms, which might have improved the soil aggregations; photo-synthetic carbon and available soil moistures (Ashworth et al., 2017). Both CA and partial CA-based scenarios showed significantly higher SOC than farmers’ practice. The SOC content of soil in CA-based systems are less prone to loss due to lower soil disturbance and SOC gets protected within soil aggregates resulting in higher SOC (Kumar et al., 2022b). Higher available macro-nutrient (N, P and K) and micro-nutrient (Mn, Zn and Cu) under CA-based scenarios could be ascribed to nutrient mobilization and mineralization caused by higher microbial activity and presence of a more labile pool of carbon (Naik et al., 2017).

The bacterial abundance was significantly affected by the several soil properties. Bacterial population and activity were positively enhanced by better carbon input, i.e., crop residue (above ground crop residue, root and rhizodepositions) in CA-based practices. The presence of copiotrophic bacteria negatively correlated with oligotrophic nature of bactera. The observed relationship among phyla is due to the occurrence of specific phyla, that are sensitives to soil condition produced by different crop management practices. Availability and presence of macro–micro nutrients showed an increase in micobial counts than an increase in total organic matter (Sharma et al., 2022). Crop residue act as the source of K, Zn, and Mn for microorganisms, and hence, promote the growth of some bacterial populations. Farmer’s based practices of conventional methods showed significantly lowest SQI value due to deterioration or degradative effect of the soil properties (Choudhary et al., 2020). Different tillage-*cum*-crop-establishment methods reported direct and indirect influences on microbial abundance and activity through changes in soil chemical, nutritional properties, and alteration in macro-organism population (Van Leeuwen et al., 2015; Choudhary et al., 2020).

## 5. Conclusion

CA-based management practices change the soil physical, biological, and chemical characteristics in rice-wheat-greengram system of eastern Indo-Gangetic Plains. Higher earthworm counts (3.6 times), soil organic carbon (11.94%), macronutrient (NPK) (14.50–23.57%), and micronutrient (Mn, and Cu) (13.25 and 29.57%) content were recorded in CA-based scenario than in tillage-intensive farmers practice. The changes in pH and status of nutrients (micronutrients) affect composition of bacterial communities in different agricultural management practices. The CA-based scenarios harbor a high abundance of *Proteobacteria* (13%) whereas conventional tillage-based scenarios were dominated by the phyla *Acidobacteria* and *Chloroflexi*. The observed relationship among the phyla is due to the occurrence of specific phyla, that are sensitive to the soil conditions produced by different crop management practices. Thus, the present study is a step foreward that provides a crucial information on impact of tillage -*cum*- crop establishment methods on soil biological, physical, and available nutrient status and their role of soil bacterial diversities and compositions in rice-wheat systems for possible implications of soil sustainability and resilience against the possible climatic risks. However, long-term studies across seasons and cropping systems are needed to draw more reliable and expected indicators to monitor the soil sustainability and resilience in the Indo-Gangetic Plains (IGP) of South Asia.

## Data availability statement

The datasets presented in this study can be found in online repositories. The names of the repository/repositories and accession number(s) can be found at: https://www.ncbi.nlm.nih.gov/genbank/, PRJNA914075.

## Author contributions

RK and AD: conceptualization, data curation, formal analysis, writing, review and editing. JC and SM: data curation, formal analysis, writing, review and editing. SN: data curation and writing eview and editing. JM and BB: investigation, methodology, project administration, and resources. SP: data curation, formal analysis, review, and editing. SrK and HH: investigation, data curation, and formal analysis. SjK: data curation and formal analysis. VK, AM, and SS: investigation, methodology, and project administration. SC: investigation, project administration, and resources. RM: methodology, project administration, and resources. PC: investigation, methodology, and resources. All authors contributed to the article and approved the submitted version.

## Funding

The present study was carried under the “Cereal Systems Initiative for South Asia” project financially supported by the Bill & Melinda Gates Foundation and the United States Agency for International Development.

## Conflict of interest

The authors declare that the research was conducted in the absence of any commercial or financial relationships that could be construed as a potential conflict of interest.

## Publisher’s note

All claims expressed in this article are solely those of the authors and do not necessarily represent those of their affiliated organizations, or those of the publisher, the editors and the reviewers. Any product that may be evaluated in this article, or claim that may be made by its manufacturer, is not guaranteed or endorsed by the publisher.

## Footnotes

^1^Available at: https://cran.r-project.org/bin/windows/base/
