# Supplementary material for: Influence of conservation agriculture-based production systems on bacterial diversity and soil quality in rice-wheat-greengram cropping system in eastern Indo-Gangetic Plains of India
Source: Front Microbiol. 2023 Jul 5;14:1181317. doi: 10.3389/fmicb.2023.1181317 (PMC10356824; doi:10.3389/fmicb.2023.1181317)
Supplement: Supplementary file 1 [file Data_Sheet_1.doc]

**Influence of conservation agriculture-based production systems on bacterial community and soil quality in rice-wheat-greengram cropping system in eastern Indo-Gangetic Plains of India**

Rakesh Kumar^a*†^, Jaipal Singh Choudhary^b^*^†^, Sushanta Kumar Naik^b^, Surajit Mondal^a^, Janki Sharan Mishra^c*^, Shis Pal Poonia^d^, Saurabh Kumar^a^, Hansraj Hans^a^, Sanjeev Kumar^a^, Anup Das^a^, Virender Kumar^e^, Bhagwati Prasad Bhatt^f^, Suresh Kumar Chaudhari^f^, Ram Kanwar Malik^d^, Peter Craufurd^g^, Andrew McDonald^h^, Sonam Rinchen Sherpa^d^

*^a^ICAR Research Complex for Eastern Region, Patna, Bihar-800 014, India*

*^b^ICAR Research Complex for Eastern Region, Farming System Research Centre for Hill and Plateau Region, Plandu, Ranchi-834 010, Jharkhand, India*

*^c^ICAR Directorate of Weed Research, Jabalpur-482 004, Madhya Pradesh, India*

*^d^Cereal Systems Initiative for South Asia (CSISA)-CIMMYT, Patna, India*

*^e^International Rice Research Institute, Los Banos, Philippines*

*^f^ICAR Natural Resource Management Division, New Delhi-110 002*

*^g^Stratigic Research Team, CIMMYT, Kathmandu, Nepal-5186*

*^h^Soil and Crop Sciences Section, School of Integrative Plant Sciences, Cornell University, Ithaca, NY, USA*

^†^*These authors contributed equally to this work and share the first authorship*

**Corresponding author:** [**rakeshbhu08@gmail.com**](mailto:rakeshbhu08@gmail.com) **(Rakesh Kumar)**

[**choudhary.jaipal@gmail.com**](mailto:choudhary.jaipal@gmail.com)**;** [**Jaipal.choudhary@icar.gov.in**](mailto:Jaipal.choudhary@icar.gov.in) **(Jaipal Singh Choudhary)**

**Supplementary Table 1.** Methods of analysis of variance (ANOVA) technique used in different analyses

| **S.No.** | **Analysis parameters** | **Mean separation method** | **Significance level** |
| --- | --- | --- | --- |
| 1. | Soil quality index (SQI) | Duncan’s multiple-range test | *p*<0.05 |
| 2. | Distribution of dominating bacterial phyla | Duncan’s multiple-range test | *p*<0.05 |
| 3. | Relative abundance at phylum, Order, and OTU level | Kruskal-Wallis test | *p*<0.05 |
| 4. | Earthworm counts and soil chemical properties | Duncan’s multiple-range test | *p*<0.05 |
| 5. | Diversity indices | Duncan’s multiple-range test | *p*<0.05 |


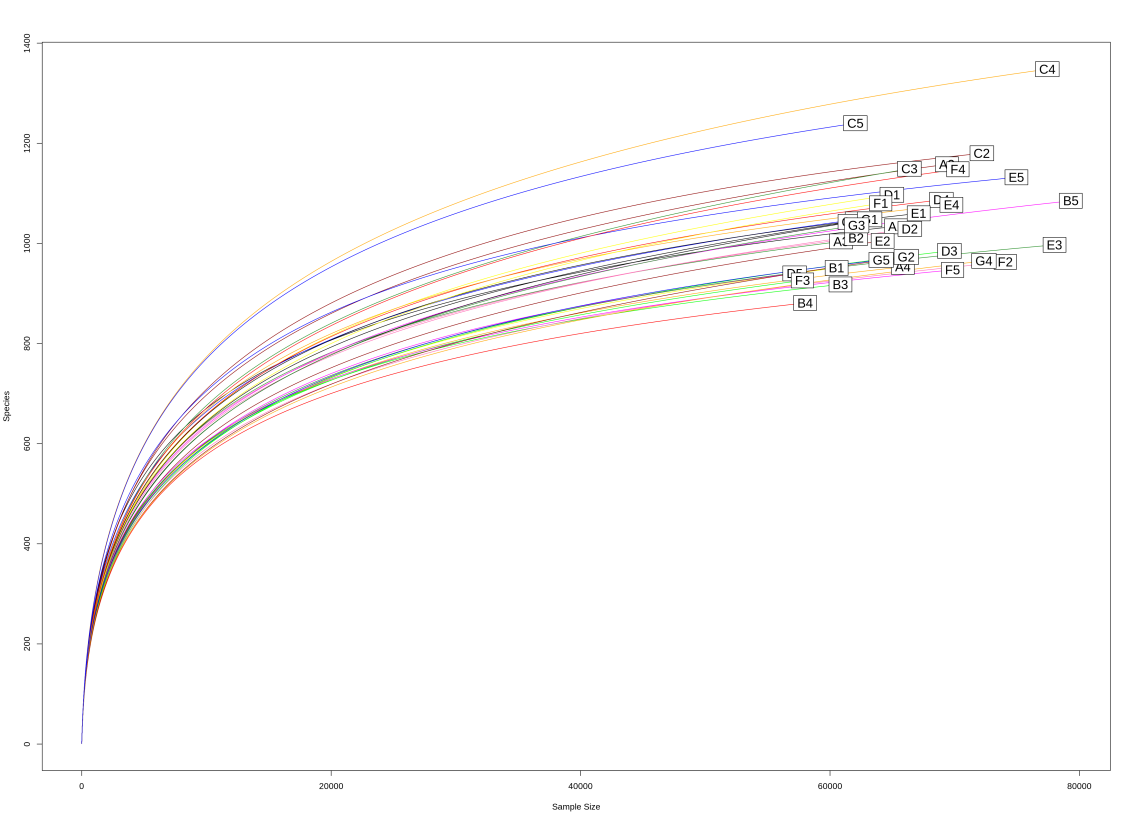


**Supplementary Figure 1**. Rarefaction curves of 35 samples collected from different scenario of agriculture change. Where curves; A1-A5 of scI; B1-B5 of scII, C1-C5 of scIII, D1-D5 of scIV; E1-E5 of scV; F1-F5 of scVI and G1-G5 of scVII scenario represents.


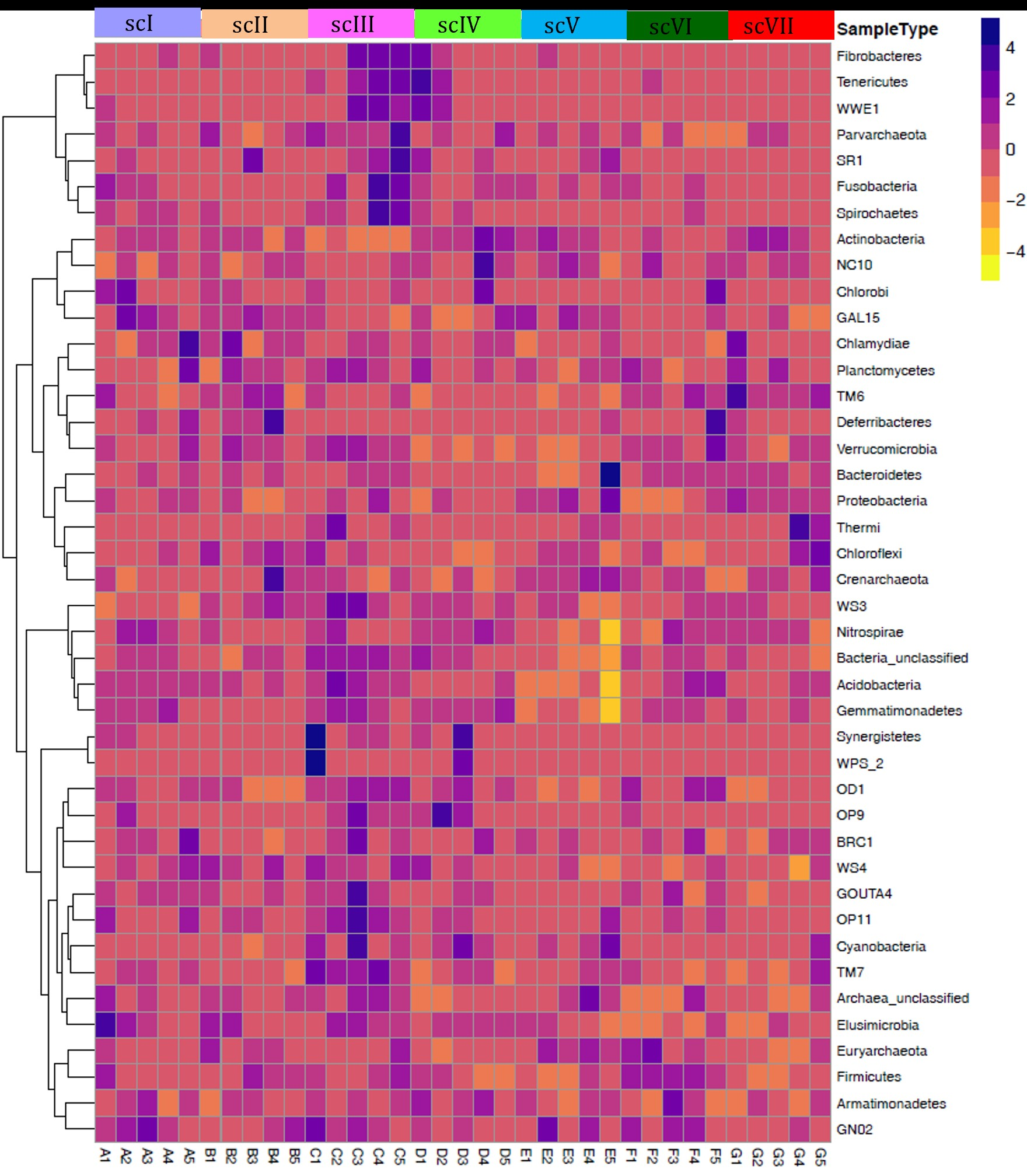


**Supplementary Figure 2.** Heatmap showing average of 16S rRNA gene OTUs between different tillage-cum-crop-establishments scenarios. The colour of heatmap indicates reactive abundance ranging from deep blue (higher) to yellow (lower abundances).
